# Supplementary material for: Identification of photoperiod-induced specific miRNAs in the adrenal glands of Sunite sheep (Ovis aries)
Source: Front Vet Sci. 2022 Jul 22;9:888207. doi: 10.3389/fvets.2022.888207 (PMC9354845; doi:10.3389/fvets.2022.888207)
Supplement: Supplementary file 1 [file Data_Sheet_1.ZIP › Supplementary file/miRDeep2 file.zip!/miRDeep2 file/Know_Structure/SP42A1/oar-mir-485.pdf]

miRBase precursor : oar-mir-485
Total read count : 989
oar-miR-485-5p read count : 360
oar-miR-485-3p read count : 628
remaining reads : 1

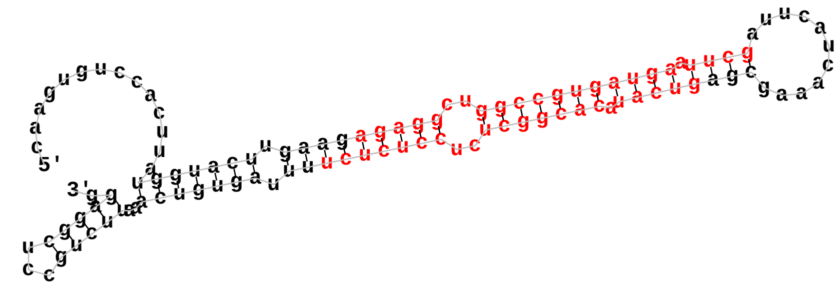

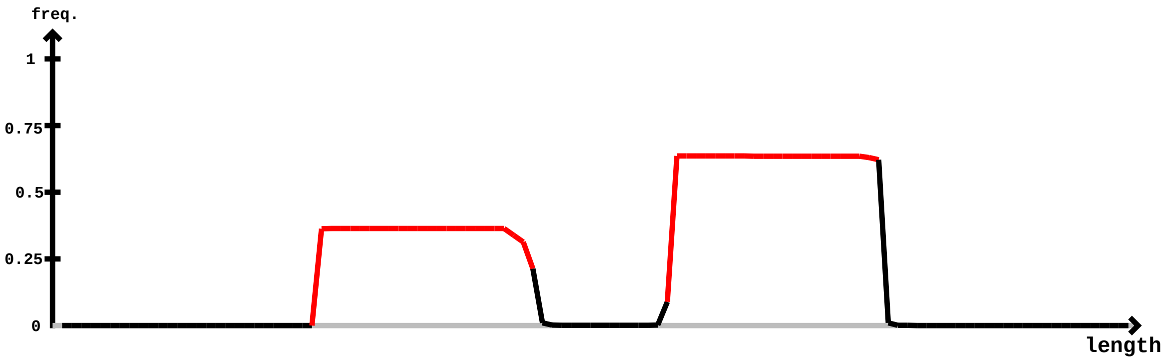


```
                                oar-miR-485-5p
                                                                        oar-miR-485-3p
5'- caaguguccacuuaugguacuugaagagaggcuggccgugaugaauucgauucaucaaagcgagucauacacggcucuccucucuuuuagugucaaauucugccucggagg   -3'    exp
    ((((((.(((....)))))))))((((((((..(((((((((((.((((...........)))))))).)))))))..)))))))).........(((((...))))).   reads    mm    sample
    ..........................agaggcuggccgugaugaau.................................................................   25       0     seq
    ..........................agaggcuggccgugaugaauu................................................................   25       0     seq
    ..........................agaggcuggccgugaugaauuc...............................................................   99       0     seq
    ..........................agaggcuggccgugaugaauucg..............................................................   201      0     seq
    ..........................agaggcuggccgugaugaauucga.............................................................   8        0     seq
    ..........................agaggcuggccgugaugaauucgau............................................................   1        0     seq
    ...........................gaggcuggccgugaugaauucg..............................................................   1        0     seq
    .................................................auucaucaaagcgagucauaca.........................................   1        0     seq
    .............................................................gagucauacacggcucuccucucu..........................   1        0     seq
    ..............................................................agucauacacggcucuccucuc...........................   1        0     seq
    ..............................................................agucauacacggcucuccucucu..........................   84       0     seq
    ..............................................................agucauacacggcucuccucucuuuu.......................   1        0     seq
    ...............................................................gucauacacggcucuccucu............................   5        0     seq
    ...............................................................gucauacacggcucuccucuc...........................   7        0     seq
    ...............................................................gucauacacggcucuccucucu..........................   520      0     seq
    ...............................................................gucauacacggcucuccucucuu.........................   9        0     seq
```
